# Effect of *Moringa oleifera* consumption on diabetic rats

**DOI:** 10.1186/s12906-018-2180-2

**Published:** 2018-04-10

**Authors:** A. Villarruel-López, D. A. López-de la Mora, O. D. Vázquez-Paulino, A. G. Puebla-Mora, Ma R. Torres-Vitela, L. A. Guerrero-Quiroz, K. Nuño

**Affiliations:** 1 Departamento de Ciencias Biomédicas, Centro Universitario de Tonalá, Universidad de Guadalajara, Av. Periférico Norte No. 555, 48525 Tonalá, Jalisco Mexico; 2 Laboratorio de Microbiología Sanitaria, Centro Universitario de Ciencias Exactas e Ingenierías, Universidad de Guadalajara, Marcelino García Barragán No. 1421, 44430 Guadalajara, Jalisco Mexico; 3 Laboratorio de Patología, Centro Universitario de Ciencias de la Salud, Universidad de Guadalajara, Sierra Mojada No. 950, 44340 Guadalajara, Jalisco Mexico; 4 Departamento de Producción Animal, Centro Universitario de Ciencias Biológicas y Agropecuarias, Universidad de Guadalajara, Camino Ramón Padilla Sánchez No. 2100, Zapopan, Jalisco Mexico

**Keywords:** Diabetes mellitus, *Moringa oleifera* powder, Glucose, Genotoxicity, Lactic acid bacteria

## Abstract

**Background:**

Therapeutic use of leaves of *M. oleifera* has been evaluated in diabetes because of its possible capacity to decrease blood glucose and lipids concentration after ingestion, as result of the polyphenols content and others compounds. Nevertheless most results have been obtain from leaf extract, therefore this study would use leaf powder as the regular way of consumption of population to know effects over toxicity glucose, triglycerides, cholesterol, corporal weight, and predominant groups of microbiota.

**Methods:**

Powdered leaf was administrated in different doses to know toxicity and genotoxicity using LD50 and micronuclei assay. Hyperglycemia was induced by alloxan on Sprague Dawley rats. Glucose and body weight were measured once a week meanwhile cholesterol and triglycerides were analyzed at the end of the study by commercial kits. Different organs were examined by hematoxylin-eosin technique. Lactic acid bacteria and *Enterobacteriaceae* were enumerated from stool samples.

**Results:**

The tested doses revealed no lethal dose and no significant differences in genotoxicity parameter. The consumption of the leaves showed a hypoglycemic effect (< 250 mg/dL in diabetic *M. oleifera* treated group), however in corporal weight showed an increased (> 30 g over no *M. oleifera* treated groups). There was no change in enumeration of lactic acid bacteria (8.4 CFU/g) but there were differences in the predominance of type of lactobacillus and enterobacteria enumeration.

**Conclusions:**

These results help to increase information over the most popular use of *M. oleifera* and its safety. However there are needed more studies over the hypoglycemic mechanisms and effects over intestinal microbiota.

## Background

Diabetes mellitus is a disease characterized by hyperglycemia caused by the impairment of insulin secretion, insulin action or both. A chronic increase in glucose levels can lead to macro- and microvascular complications, such as heart disease, hypertriglyceridemia, nephropathy, and neuropathy. In diabetes mellitus 2, insulin resistance can also lead to gastroparesis as a secondary effect, causing abdominal pain, nausea, emesis, meteorism, and an alteration in intestinal microbiota marked by enterobacteria predominance and a decrease in the number of beneficial bacteria [1, 2].

Glycemic control through diet is necessary for preventing or limiting the consequences of diabetes mellitus. Therefore, the consumption of functional foods and nutraceutical or bioactive compounds derived from plants used as food can be used as nutritional tools because of their clinical effects. For example, the ingestion of *Moringa oleifera* could bring biological benefits due to the nutritional content of its leaves, such as protein, glucoside, vitamins (A, C), and minerals [3, 4].

*Moringa oleifera* is an Indian tree that has been cultivated in diverse regions of Mexico, and it is referred to as “drum stick tree” or the “horse riding tree.” It belongs to the family *Moringaceae,* the order *Brassicales,* and the genus *Moringa,* which contains 13 species ranging in height from 5 to 10 m. It has an open crown of drooping, feathery foliage, flowers with distinctive green patches at the tips of the petals and sepals, tripinnate leaves and trunk. This tree is important because its flowers, pods, and leaves have medicinal uses. It has been reported that the flower contains a stimulant and is used to treat inflammation; the spots and seeds have liver-protective and antihypertensive properties, while the leaves have been used to treat microbial infections and to control glucose levels.

The leaves are eaten as vegetables of food ingredient because of the high content of vitamins, antioxidants and macronutrients to improve nutritional deficiencies [5]. However the study of effect of biological compounds of different part of *Moringa oleifera* plant have brought different action mechanism, functional benefits and toxicity profile that have not yet been elucidated. Is in that reason that it has been suggested that the use of new pharmaceutical and nutraceutical products, as *M. oleifera,* must be tested first for safety in appropriate in vitro and in vivo models, before being used in human health, in order to ensure the absence of toxicity and for better understanding of action mechanisms [6, 7]. In spite of nutraceutical beneficial properties, the different compounds of the plant present distinct pharmacological effects, including toxicity profiles, which have not yet been completely elucidated. Also, international regulations relating to human health demand that all kind of pharmaceutical and nutraceutical products are tested for their safety, and the way to ensuring this is to conduct toxicity tests in appropriate in vitro and in vivo models [5]. Therefore for toxicological evaluation it has been used animal models to reveal histopathological damage [8].

There have been used in biological assay aqueous and ethanol extract of leaf in different doses, meanwhile leaf powder studies have been most done in clinical research [9]. Thus it can be use in vivo models to bring more information about powder leaf consumption effect on different diseases. For example, induction of experimental diabetes in rats is a convenient model to study activity of hypoglycemic agents over hyperglycemia and it is consequences where it has been observed the possible antioxidant and antidiabetic effects through plasma glucose, triacylglycerol and cholesterol monitoring, microscopic lesion observation, marker enzyme, serum and lipid peroxidation measuring and for understanding the pathophysiology [9, 10]. Also the experimental animal model of diabetes mellitus can be done by chemical induction using streptozotocin or alloxan which diabetogenic action has been employed and proven in different animal species, with different rout of administration or nutritional stratus [11]. Humans and mouse genotype is very similar between them, this influences metabolism, as it becomes highly comparable with humans, and different strains of mice differ in their response to high fat diet and sensitivity to metabolic diseases. *BALB*/c mice are naturally resistant to the development of high fat diet induced obesity and development of diabetes as a result [12]. Mouse is a good biological tool that allows the analyses of different tissues with little limitation on the amount of biological materials available. This animal model provide an important tool for study of the pathogenesis, prevention and treatment of diabetic complications [13]. Mouse is economical and requires simple husbandry compared to larger mammals, and there is a huge volume of literature on the physiology, behavior, and biochemistry of such rodents. Importantly, it is possible to modify the diet of mouse or treat them with drugs to mimic specific diseases and/or to improve their health status [14].

There have been used in biological assay aqueous and ethanol extract of leaf in different doses, meanwhile leaf powder studies have been most done in clinical research [15]. Thus it can be use in vivo models to bring more information about powder leaf consumption effect on different diseases. For example, induction of experimental diabetes in rats is a convenient model to study activity of hypoglycemic agents over hyperglycemia and it consequences where it have been observed the possible antioxidant and antidiabetic effects through plasma glucose, triacylglycerol and cholesterol monitoring, microscopic lesion observation, marker enzyme serum and lipid peroxidation measuring [15, 16].

The therapeutic use of *M. oleifera* leaves has been evaluated in diabetes because of their possible capacity to decrease blood glucose concentrations after ingestion because they contains polyphenols such as quercetin-3-glycoside, rutin, kaempferol and glycosides [17–19]. Decrease in blood sugar because of *M. oleifera* therapy can be noticed in different tests as: fasting blood glucose, oral glucose tolerance test and post prandial glucose on diabetic rats, in an average decrease of 25% or more [20]. The antidiabetic activity of *Moringa* seed powder has been observed in rat models with the decreased glucose and the amelioration of levels of lipid peroxide, the diminish levels of IL6, and immunoglobulins A in comparison with diabetic positive control in both insulin resistant and insulin deficient bioassays [21, 22]. Meanwhile Anudeep et al.*,* showed the *Moringa oleifera* contain soluble fiber that enhance amelioration of levels of glucose, proliferation of lymphocytes and induced nitric oxide from macrophages. In other study observed the fortification of *M. oleifera* in diabetes can lead to fasting blood glucose dropped, which can help to reduce the entrance of glucose to mitochondria and diminish the release reactive oxygen species and advance glycated end products (AGEs) which can enhance cell adhesion and inflammation in diabetic patient [23]. Treatment with *Moringa* has showed after histological examination of pancreas from diabetic rats, a significantly damage reversed in the histoarchitectural of the islet cells [24].

Elevation of lipid concentration is part of the physiopathology of diabetes mellitus which increased the risk of premature arterosclerosis. Therefore the use of *M. oleifera* is clamed to possess hypolipidemic properties. It was found that crude leaf extract along with high-fat diet decrease cholesterol and triacylglycerol in serum [26]. When bioassay was done with *Moringa* in comparison with lovastatin, it was found that both decreased the lipid profile on liver, heart and aorta [25]. In other study it was observed that consumption of *M. oleifera* for 15 weeks can decrease total cholesterol, triglycerides, LDL-cholesterol, atherogenicity index in HIV negative patients [26].

However most results have been obtained in studies of leaf extracts; therefore, this study investigates the use of leaf powder, which is the common method of consumption, to investigate the effects on glucose, triglycerides, cholesterol and corporal weight and the predominant groups of microbiota. To expand the knowledge on biological properties of *Moringa oleifera* powder leaf in this study clinical and toxicological effect on alloxan-induced diabetic rats were assessed, specially to know variability in acute toxicity and microbiota impact using one route of administration the is intended in human.

## Methods

### Plant material

*Moringa oleifera* leaves were collected from a private herbarium at the Universidad Autónoma de Sinaloa, Sinaloa, México, with registration code UAS-MO-10, Lot Number 5. The leaves were washed and oven dried at 40 °C for 24 h, until the moisture content reached 10%. The dry leaves were pounded, and the powder was kept at room temperature until use [27].

### Lethal dose 50 and genotoxicity

The lethal dose 50 was determined in Balb-C56 male mice by the administration of different doses of leaf powder (mg/kg) according to Lorke (1983) [28], with a modification in the number of animals (*n* = 5) according to Mann-Whitney U chart to get the two tails critical value with the minimal animal number use. There were four randomly assigned groups (5 mice/group/per cage): Group 1: control group (saline solution); Group 2: administration of 100 mg/kg of *Moringa oleifera;* Group 3: 200 mg/kg of *M. oleifera*; and Group 4: 500 mg/kg of *M. oleifera.*

Animals were under observation for signs and symptoms of toxicity or death from 24 h until 7 days after the administration of *M. oleifera* to determine the lethal dose. To investigate genotoxicity, a day 7 blood sample was taken from the tip of the tail of each animal, and a peripheral blood smear was performed on the samples. The blood smears were dried at room temperature, fixed with absolute ethanol for 10 min, and stained with acridine orange for analysis. Erythrocytes were micronucleated according to the procedure detailed by Zúñiga-González et al. [29]. All samples were enumerated manually without previous knowledge of group assignment. Observations were performed with a BX51 microscope with epifluorescence using a 100X immersion objective. Cumulative damage was determined by quantifying the frequency of micronucleated erythrocytes (MNEs) over 10,000 total erythrocytes (TEs), composed of polychromatic erythrocytes (PCEs) and normochromatic erythrocytes (NCEs). To determined recent damage, mononucleated polychromatic erythrocytes (MNPCEs) over 1000 PCEs were counted. The proportion of PCEs/1000 TEs was quantified to determine the cytotoxic potential of the diet [30].

### Induction of hyperglycemia

Hyperglycemia was induced by applying 150 mg/kg alloxan monohydrate (A7413, Sigma-Aldrich®, St. Louis, MO, USA). After 72 h, a blood test confirmed hyperglycemia (> 200 mg/dL) [31], after which *M. oleifera* was orally administered.

### Experimental design

In the present study, thirty healthy male Sprague Dawley rats with an average body weight of 180–200 g were used in a special cage with water ad libitum and 12 h light/dark cycle in a room temperature controlled at 25 °C. The animals were distributed into 5 groups (*n* = 6), which included one control group, one healthy group treated with *M. oleifera*, one untreated diabetic group, one diabetic group treated with *M. oleifera*, and one diabetic group treated with glibenclamide (Sigma-Aldrich®, St. Louis, MO, USA). The administered dose of *Moringa oleifera* was 50 mg/day, and the glibenclamide dose was 600 μ/kg/day [32]. Both were administered orally for 8 weeks using a cannula needle (VWR, 20068–642, West Chester, PA, USA) at the laboratory. Animals were wuthanized by exsanguination after isoflurane anesthesia.

### Clinical parameters

Body weight (g) and glucose were measured weekly using a triple-arm scale (730-SW, Ohaus, Pine Brook, NJ, USA) with a basket to hold the animals and an Accu-Check Active® (Roche Diagnostics®, Indianapolis, IN, USA; range 10–600 mg/dL), respectively. At the end of the experimental period, a 12-h fast was imposed on the animals, after which they were sacrificed. Triglycerides (11503), cholesterol (11505), LDL (11585) and HDL (11557) were measured with commercial kits (BioSystems® S.A, Barcelona).

### Histopathology

At the end of the biological assay, different organs, including the intestine, liver, and kidney, were extracted and placed in a sterile container. Organs were placed in 10% neutral buffered formalin (HT501128, Sigma-Aldrich®, St. Louis, MO, USA), sectioned for histological analysis, stained with hematoxylin-eosin, and examined using light microscopy. Diagnosis of abnormalities was based on histological examination of the biopsies. For this purpose, the animals were sacrificed using CO_2_.

### *Lactic acid bacteria* (*LAB*) and *Enterobacteriaceae*

Lactic acid bacteria and *Enterobacteriaceae* were enumerated in 1 g stool samples. Briefly, 9 ml of 0.85% sodium chloride (Promega H5271, Madison, WI, USA) was added to the sample and homogenized. Three additional decimal dilutions (1:10, 1:100, and 1:1000, *v*/v) were performed. For LAB enumeration, a 0.1 ml sample was taken from each dilution, placed on an agar plate containing MRS medium (Man, Rogose and Sharpe, BD Difco Laboratories®, Sparks Maryland, MD, USA), and cultured using the spread plate method with an incubation at 35°C for 48 ± 2 h in a candle jar, under an approximately 15% oxygen atmosphere [33]. Three replicate plates were prepared for each dilution. Colonies were counted after incubation. Gram-positive colonies, which were catalase negative and oxidase positive, were isolated, purified, subcultured on MRS agar and treated as presumptive LAB. The isolates were characterized and identified using API 50CH and API 50CHL medium (bioMe’rieux®, Marcy-l’E’toile, France) following the manufacturer’s instructions.

The *Enterobacteriaceae* were enumerated in the samples from this solution and placed on five different media: eosin methylene blue agar (EMB), MacConkey, brilliant green agar (BG), Salmonella–Shigella agar (SS), and Hektoen enteric agar. After incubation at 35 °C for 24 h, representative colonies were isolated, purified, and subcultured on selective agar. They were then subjected to Gram staining.

### Statistical analysis

Differences between treatments were identified with an analysis of variance (ANOVA; SPSS-PC, version 20.0). In cases where the standard deviation differed between groups, *Mann-Whitney U* and *Kruskal Wallis* multiple comparison nonparametric tests were applied with a 95% (*p* < 0.05) confidence level.

### Ethical considerations

All procedures performed in studies involving animal models were in accordance with the ethical standards of the institutional ethics committee of Centro Universitario de Tonalá and the Mexican Official Standard NOM-062-ZOO-1999, which were approved by the same committee.

## Results

### Toxicity

**Fig. 1:**
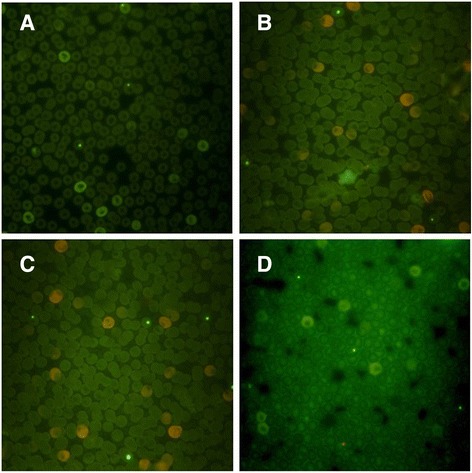
Micronucleus test of Control Group with and without *Moringa oleifera*. **a** Control Group, water. **b** *M. oleifera* Group, 100 mg. **c** *M. oleifera* Group, 250 mg. **d** *M. oleifera* Group, 500 mg. No significant changes was observed in all smears between control Group and *M. oleifera* treatment

The tested doses of *M. oleifera* revealed no adverse effects in the experimental animals, including possible alterations such as food intake, unusual body growth, reduced activity, diarrhea, bleeding, or death. Therefore, no lethal dose was determined. As for genotoxicity, it was observed that the control group showed no significant difference in any genotoxicity parameter after *intra* and *inter* group comparisons of MNEs, MNPCEs and PCEs, as expected (Fig. 1). *M. oleifera* treatment groups did not show differences (*p > 0.*05) between the various doses (100, 250 and 500 mg/kg) in MNEs. A slight rise was seen in MNEs compared to the control group, but the difference was not significant (*p* > 0.05). *Intra* and *inter* group comparisons of MNPCEs detected no significant (*p >* 0.05) difference between doses of *M. oleifera* or between treatment groups and the control group. PCE values, similar to previous groups, showed no statistically significant difference (*p >* 0.05) between *intra* and *inter* group doses or between treatment groups and the control group (Table 1).

**Table 1:** MNE, MNPCE and PCE frequencies (%)

	Control Group (*n* = 5)			*Moringa Oleifera* Group (*n* = 5)		
Dose (mg)	MNE	MNPCE	PCE	MNE	MNPCE	PCE
100	64.0 ± 23.02	0.8 ± 0.44	22.2 ± 2.68	94.0 ± 45.60	0.8 ± 0.83	25.8 ± 11.18
	*NS	*NS	*NS	*NS	*NS	*NS
	*NS	*NS	*NS	*NS	*NS	*NS
250	58.0 ± 40.86	0.40 ± 0.54	32.2.0 ± 20.8	38.0 ± 17.16	0.20 ± 0.44	22.8 ± 22.83
	*NS	*NS	*NS	*NS	*NS	*NS
	*NS	*NS	*NS	*NS	*NS	*NS
500	66.0 ± 27.01	1.2 ± 1.30	23.8 ± 2.48	110 ± 81.85	1.2 ± 1.09	29.4 ± 4.76
	*NS	*NS	*NS	*NS	*NS	*NS
	*NS	*NS	*NS	*NS	*NS	*NS

*NS not statistically significant

Data are expressed as the mean ± standard deviation per group. *Moringa* solution was administered interrogatorily to rats. MNE: micronucleated erythrocytes/10000 TE; MNPCE: micronucleated polychromatic erythrocytes/1000 PCE; PCE: polychromatic erythrocytes/1000 TE; TE: total erythrocytes; n: simple size, 5 rats per group; NS: not significant. *: intra-group significance (*p* > 0.05) (repeated measures ANOVA and LSD test post hoc for multiple comparisons); **: inter-group significance (*p* > 0.05) (one-way ANOVA and Dunnett t-test post hoc for multiple comparisons).

### Lethal dose

The doses of *M. oleifera* powder leaf tested reveled no adverse effects in experimental animals, including possible alterations such as food intake, unusual body growth, reduced activity, diarrhea, bleeding or death.

### Histopathology

Intestinal tissue sections (38 × 0.6 × 0.6 cm) showed the control group and the healthy group treated with *M. oleifera* to have intestines lined by intestinal cylindrical epithelium forming glands, lamina propria with a transmucosal lymphoplasmacytic inflammatory infiltrate, and regular lymphocytes with no histopathological indicator of metaplasia, dysplasia, or malignancy.

**Fig. 2:**
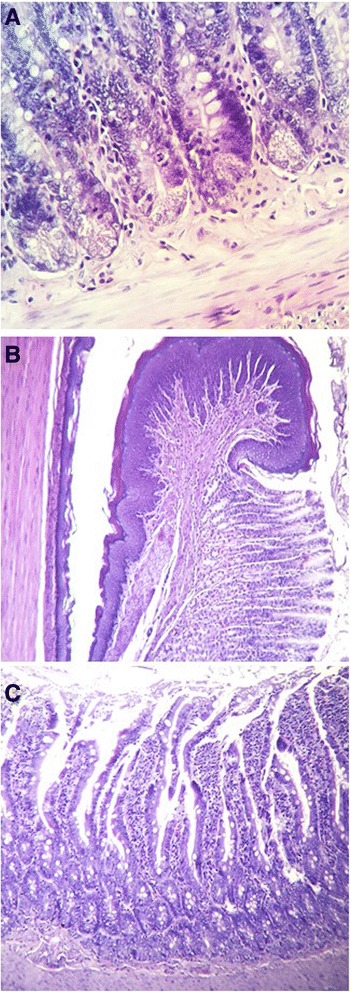
Control group with *Moringa oleifera* treatment. **a** Normal architecture of the large intestine. **b** Normal gastroesophageal union was observed. **c** No changes in small intestine

All diabetic groups were shown to have an epithelium with severe atrophy covering large areas, flattened villi, inflammation, and a lamina propria with lymphoplasmacytic inflammatory infiltrate negative for malignancy. One untreated diabetic rat showed necrosis (Fig. 2).

### Clinical parameters

**Fig. 3:**
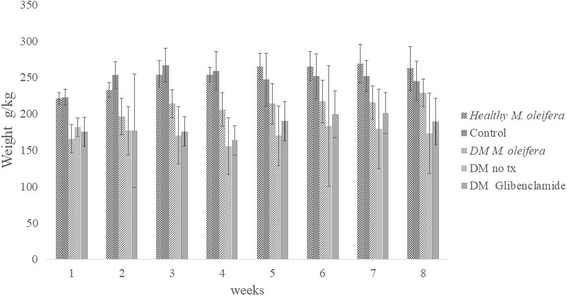
Weight (g/kg) of study groups during 8 weeks. Data are expressed as mean ± standard deviation *per* group

The initial body weight for all groups was ±200 g. After alloxan induction, diabetic groups decreased in weight. During the 8 weeks, the body weight of the control group was lower than that of the healthy group treated with *M. oleifera* (246 g vs 263 g, respectively), but there was no significant difference. The diabetic group treated with *M. oleifera* showed an increased body weight in comparison with both the diabetic group treated with glybenclamide and the untreated diabetic group (229 g, 190 g, and 173 g, respectively) (Fig. 3). The diabetic group treated with *M. oleifera* was different than the untreated diabetic group (*p <* 0.05).

**Fig. 4:**
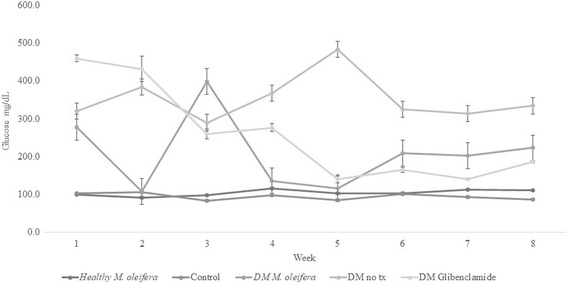
Glucose values (mg/dL) in study groups during experiment. Data are expressed as mean ± error standard *per* group

Glucose levels of 100 mg/dL were measured in the experimental animals at the beginning of the study (*p* > *0.05*). After hyperglycemia induction, the diabetic groups showed glucose values of ±300 mg/dL (*p* > *0.05*). In the second week, glucose levels in the diabetic group treated with *M. oleifera* diminished in comparison to the untreated diabetic group. On the other hand, the control group and the healthy group treated with *M. oleifera* did not show differences over this time period (*p > 0.05*). Triacylglycerol values were not different between diabetic groups (Fig. 4). The healthy group treated with *M. oleifera* showed lower values (24 mg/dL) in comparison to the control group (53 mg/dL).

### *Lactic acid bacteria* (LAB) and *Enterobacteriaceae*

There were not significant differences between study groups in LAB enumeration (8.4 UFC/g). The predominant genus identified in the control group, the healthy group treated with *M. oleifera*, and the diabetic group treated with *M. oleifera* was *Lactobacillus fermentum.* On the other hand, the untreated diabetic group and the diabetic group treated with glyburide revealed the presence of *Lactobacillus acidophilus* and *Leuconostoc lactis*. Enterobacteria enumeration produced lower counts in the healthy group treated with *Moringa oleifera* in comparison to the other diabetic groups (*p < 0.05*).

## Discussion

*Moringa oleifera* possesses bioactive compounds, the qualities of which have been studied in recent years to establish a more scientific basis for its use and to elucidate its biological activity. The leaves have been used as antidiabetic, antibacterial, and anti-inflammatory herbal drugs [21, 34–38]. There are studies that show no risk in using *M. oleifera* leaves at various doses; however, most of these data have been derived from studies on plant extract. Therefore, the study of leaf powder consumption brings new knowledge about the safety of this plant while providing options for plant preservation without the loss of nutrients, especially since the leaf of this plant can be used as a vegetable in soup preparations, cooked and mixed in with ground peanut cake or packaged in powder pills [21, 36–38].

Furthermore, *M. oleifera* is a source of antioxidants, vitamins, and a protease-resistant glycoprotein that functions as dietary fiber [39–41]. *Moringa oleifera* has antioxidant activity because it contains phenolic compounds and flavonoids, specifically three classes of phytochemicals: glucomoringin, flavonoids (quercetin and kaempferol) and phenolic acids (chlorogenic acid) [41]. These bioactive compounds can exert antioxidant and anti-inflammatory effects that could induce cellular protection [42, 43], as can be observed in *M. oleifera* regulation of the formation of micronuclei in response to damage to the genetic material in cells. Micronuclei are chromosomal fragments or entire chromosomes that were not included into the daughter cell nuclei at mitosis. The erythrocyte micronucleus assay is a simple and minimally invasive method that detects in vivo structural or numerical chromosome damage [5]. The results of this study show that *M. oleifera* regulates the formation of micronuclei, maintaining the basal values through an antioxidant effect that neutralizes free radicals that would otherwise affect DNA. Moreover, *M. oleifera* did not increase MNE frequency and maintains the ratio of PCEs and MNPCEs. In fact, these values were the same as the values in the control group. The doses tested in this study did not reach the LD_50_, and there was no observed histopathological damage in different organs. Previous studies have shown no toxicity or adverse effects in body organs for aqueous leaf extract in rats [36, 44]. However, Assare et al.*,* (2012) [45] observed that supra-supplementation with 3000 mg/kg of aqueous leaf extract reduced albumin serum levels and total protein. Differences in results for aqueous extracts could stem from differences in extraction and purification processes or source location of the plant. Therefore, these new findings based on leaf powder, as well as previous results for leaf extract, could help to provide an enhanced understanding of the acute and chronic toxicity of *Moringa oleifera.*

Hypoglycemic activity in the bioassay could be a result of phytochemicals, which are bioactive substances with antioxidant activity that have been associated with a protective effect against chronic degenerative diseases. These hypoglycemic effects have been tested with doses of seed powder (50–100 mg/kg body weight) in diabetes mellitus, where in decreases in fasting blood sugar and serum hemoglobin A_1c_ compared with positive controls have been observed [46]. Previous studies suggest that kaempferol stimulates glucose uptake in the rat soleus muscle via the PI3K and PKC pathways. Orally administered kaempferol significantly decreased fasting blood glucose and serum HbA1c levels while improving insulin resistance. Quercetin inhibits the transport of fructose and glucose by GLUT2 in the brain and stimulates GLUT4 translocation and expression in skeletal muscle [27, 47]. This could explain the tendency towards lower blood glucose levels in the diabetes group treated with *Moringa oleifera* compared to the positive control group in the present study. Furthermore, the effect of leaf consumption on triglyceride levels showed a tendency to decrease in healthy animals, which is similar to other studies in which *M. oleifera* has been shown to have hypolipidemic potential [28].

Diabetic *M. oleifera* treated rats increased in body weight. This result is consist with previous studies. Olayaki et al., (2015) observed that oral administration of extract of *M. oleifera* significantly reduces blood glucose concentration and inhibits weight loss in alloxan-induced diabetic rats. Another previous study showed a significant increase in the body weight of rats treated with 50 mg and 100 mg of seed powder [46]. The increase in weight may be due to the content of seed powder, specifically essential amino acids and vitamins A, B, C and E. In addition, antioxidants and antimicrobial compounds (phenols, tannins, alkaloids and cumarins) can act as growth promoters [48].

Consumption of *M. oleifera* leaves did not show differences in lactic acid bacteria enumeration, although there was an effect on enterobacteria enumeration in the healthy group treated with *M. oleifera*. It has been reported that *M. oleifera* may have antimicrobial activity against *Staphylococcus aureus, Bacillus subtilis, Escherichia coli, Pseudomonas aeruginosa, Salmonella typhi, Aspergillus niger,* and *Candida albicans.* Additionally, less antimicrobial activity has been observed against Gram-positive than Gram-negative microorganisms [17]. *M. oleifera* leaves contain biocomponents whose antibacterial potentials are comparable to that of the antibiotic oxytetracycline against gram-negative organisms, which may be the result of the presence of natural antioxidants (ascorbic acid, flavonoids, phenolics and carotenoids, and even because off the presence of saponin, tannic, phenolic and alkaloid phyto constituents) [48]. Some of these compounds can lead to cell membrane perturbations and exert a β-lactam action on the transpeptidation of the cell wall. Leaf extract contains small peptides that can increase the permeability of cell membranes or cell walls. Some compounds may interact with the lipid bilayers in cell membranes, leading to the separation of the two membranes, thus leading to cellular swelling and cell death [49].

Leaf administration needs more scientific basis. Most studies of therapeutic use of *M. oleifera* are with aqueous, hydroalcohol or alcohol leaf extract against hyperlipidemia and hyperglycemia. Nerveless studies of the use of leaf powder are limited. There are reports of high-fat diet containing *Moringa* leaf powder to diminish lipid profile in dyslipidemic rats [26], over glucose tolerance in Wistar rats and Goto-Kakizaki rats modeled type 2 diabetes [48], as antioxidant or supplement as nutrition counseling [49]. However diabetes mellitus is a disease which leads to raised level of glucose, triglycerides, corporal weight and a gut microbiota disbiosis, therefore this study would bring information about relationships between leaf powder *M. oleifera* consumption in different doses with gut microbiota changes and clinical parameters.

## Conclusion

The findings suggest that consumption of *M. oleifera* powder leaves could be beneficial in the diabetes mellitus rat model over glucose values and enterobacterias enumeration. However further research will be needed to evaluate the mechanisms of action over lipids and intestinal microbiota in diabetes mellitus to increase possible uses of *M. oleifera* in functional foods as a nutraceutical.

Therefore, the study about leaf powder consumption can bring new knowledge about it safety and as an option of plant preservation without loss the nutrient, especially when leaf of this plant can be use as vegetable in soup preparation, cooked and mixed with grounded groundnut cake or in powder pills.
